# Pre-gravid body mass index is associated with a higher risk of gestational hypertension in singleton pregnancy following frozen-thawed embryo transfer

**DOI:** 10.3389/fendo.2023.1258530

**Published:** 2023-10-16

**Authors:** Lijuan Fan, Na Li, Xin Mu, Pengfei Qu, Juanzi Shi

**Affiliations:** ^1^ Assisted Reproduction Center, Northwest Women’s and Children’s Hospital, Xi’an, China; ^2^ Translational Medicine Center, Northwest Women’s and Children’s Hospital, Xi’an, China

**Keywords:** body mass index, gestational hypertension, frozen-thawed embryo transfer, obstetric outcomes, assisted reproduction technology

## Abstract

**Introduction:**

Although it is well-known that obesity increases the risk of gestational hypertension (GH) in both spontaneous and assisted reproductive technology (ART) pregnancies. Recent data show that, in ART pregnancies, frozen-thawed embryo transfer (FET) is associated with an even higher risk of GH compared with fresh transfer. However, the relationship between pre-gravid body mass index (BMI) and GH in FET pregnancies has seldom been reported.

**Objective:**

The aim of this study is to examine the effect of pre-gravid BMI on GH in singleton pregnancy following FET.

**Methods:**

A retrospective cohort study at a tertiary hospital, including a total of 7,502 women who achieved singleton pregnancy after FET, was included. All patients were enrolled only once. On the basis of the BMI definitions of the Working Group on Obesity in China (WGOC) and the World Health Organization, the women were divided into normal BMI, overweight, and obese groups. The main outcome was GH, and the effect of pre-pregnancy BMI on GH was assessed by generalized linear model.

**Results:**

The risk of GH in our study population was 6.15%. According to the BMI definitions of the WGOC, the risk of GH in the obese group (15.55%) was significantly higher than that of the overweight group (8.26%, *P* < 0.001) and the normal BMI group (4.68%, *P* < 0.001). Pre-gravid overweight and obesity were associated with higher GH risk (OR, 1.77; 95% CI, 1.41–2.20; *P* < 0.001; OR, 3.69; 95% CI, 2.77–4.91; *P* < 0.001). A non-linear relationship between pre-gravid BMI and GH was observed. The risk of GH decreased with pre-gravid BMI level up to the turning point of BMI = 28.6 kg/m^2^ (OR, 1.16; 95% CI, 1.12–1.21; *P* < 0.001).

**Conclusion:**

Pre-gravid overweight and obesity are associated with higher GH risk among singleton pregnancy following FET. Before the turning point of BMI = 26.8 kg/m^2^, the risk of GH may increase 16.4% with each one-unit increment of maternal BMI. Women preparing for FET should maintain a normal BMI to lower the chances of GH.

## Introduction

Hypertensive disorders of pregnancy (HDP) include gestational hypertension (GH) and pre-eclampsia (PE) (1), and their prevalence rates range from 1.8% to 4.4% and 0.2% to 9.2%, respectively, in all pregnancies (2). GH was defined as *de novo* hypertension after 20 weeks of gestation in the absence of proteinuria (2, 3). Women with GH are likely to develop placental abruption, disseminated intravascular coagulation, cerebral hemorrhage, and hepatic and renal failure (4). Moreover, 46% and 9.6% of pregnant women with GH progress to mild preeclampsia and severe preeclampsia, respectively (5).

It is widely known that higher pre-gravid body mass index (BMI) and assisted reproductive technology (ART) are both risk factors for GH (6). In ART pregnancies, the incidence of GH was higher in women with overweight (OR, 1.36 to 2.2) and obesity (OR, 3.17 to 4.4) as compared with women with weight (7, 8). It is important for women with overweight and obesity to lose weight before ART.

Embryos are transferred to the endometrium prepared by either fresh embryo transfer or frozen-thawed embryo transfer (FET). The application of FET has dramatically increased worldwide over the past decade because of its advantages, such as storage of excess embryos, lower incidence of ovarian hyperstimulation syndrome (OHSS), and fertility preservation. Several studies have suggested that FET is associated with higher risks of GH as compared with fresh embryo transfer (9–11). Moreover, in FET cycles, obesity is independently associated with a 1.76- to 2.25-fold greater risk for PE and eclampsia (12). Overweight, which was between normal BMI and obesity, is composed of a large proportion of infertility population. According to the 2002 National Survey, 24.5% of women of childbearing age were overweight and 23.0% were obese (13). However, few study reported the incidence of GH in women with overweight who achieved singleton pregnancies after FET. Moreover, whether there is a dose–response relationship between pre-gravid BMI and GH in FET pregnancies was unknown. Therefore, we conducted a 5-year retrospective cohort study among women with normal BMI, overweight, and obesity who achieved singleton pregnancies after FET to evaluate the effect of pre-gravid BMI on GH.

## Methods

### Study design

This was a retrospective cohort study, in which *in vitro* fertilization (IVF) was performed between January 2018 and December 2022 at the Center for Assisted Reproductive Technology of Northwest Women’s and Children’s Hospital (Xi’an, China). The protocol of the study was approved by the institutional review board of the hospital (no. 2022007). Data were extracted from electronic medical records.

The inclusion criteria were as follows: maternal age at oocyte pickup (OPU) ≤40 years, FET cycle, and achieved singleton ongoing pregnancy (presence of at least one fetal heart pulsation on ultrasound beyond 20 weeks). All participants were enrolled only once. The exclusion criterion was chronic hypertension before pregnancy.

### Body mass index assessment

Nurses measured and recorded the weight and height of all women after the initial consultation. The BMI was measured at the initial IVF consultation and calculated as kg/m^2^.

Pre-gravid BMI was categorized and analyzed according to the definitions of the Working Group on Obesity in China (WGOC) (8, 14, 15) and the World Health Organization (WHO), respectively. According to the WGOC, BMI groups were defined as follows: normal weight (BMI, <24.00 kg/m^2^), overweight (BMI, 24.00–27.99 kg/m^2^), and obesity (BMI, ≥28.00 kg/m^2^). All women were categorized into three groups. On the basis of the WHO criteria, BMI groups were defined as follows: normal weight (BMI, 18.5–24.9 kg/m^2^), overweight (BMI, 25–29.9 kg/m^2^), and obese (BMI, ≥30 kg/m^2^).

### Controlled ovarian stimulation and vitrified cryopreservation

Ovarian stimulation (OS) protocols included gonadotropin-releasing hormone (GnRH) agonist protocol, GnRH antagonist protocol, and other protocols (progestin-primed OS protocol and natural protocol). Recombinant human chorionic gonadotropin (hCG; OVIDREL; Merck Serono, Darmstadt, Germany) or GnRH-a (Decapeptyl; Ferring, Saint-Prex, Switzerland) was administered to women when two leading follicles reached 18 mm in diameter. Oocyte retrieval was performed at 36 h after recombinant hCG or GnRH-a triggered by transvaginal ultrasound–guided aspiration. Insemination method was selected according to the sperm count after sperm preparation. A morphologic score of day-3 cleavage-stage embryo was given on the basis of the number of blastomeres, the homogeneous degree of blastomeres, and the degree of cytoplasmic fragmentation, which has been extensively described in our previous study (16). If a couple has two or more high-quality cleavage-stage embryos on day 3 of embryo culture, then the embryos were selected and cultured to blastocyst stage. Blastocyst evaluation was performed according to the Gardner’s grading system (17).

For women who underwent GnRH agonist protocol and GnRH antagonist protocol, embryos were transferred into the uterus of women without OHSS, hydrosalpinx, intrauterine adhesion, and high progesterone level (>1.5 ng/mL) on the day of triggering, and, then, the spare embryos were cryopreserved for the next FET. Women who underwent Progestin-primed ovarian stimulation (PPOS) protocol had to freeze all their embryos. The vitrified cryopreservation was conducted according to standard protocols, as previously described (18).

### Frozen-thawed embryo transfer and luteal support

In this study, three different FET regimens were non-selectively and consecutively recorded. The participants were allocated to artificial cycle (AC) FET protocol, natural cycle (NC) FET protocol, or OS FET protocol according to the experience of the physician and patients’ characteristics.

Women under the NC FET protocol underwent transvaginal ultrasound on days 8 to 10 of the menstrual cycle. The day of ovulation was confirmed by transvaginal ultrasound and serum-luteinizing hormone. Up to two cleavage-stage or blastocyst-stage embryos were thawed and transferred 3 or 5 days after ovulation, respectively.

Women under the OS FET protocol started their transvaginal ultrasound and OS on the fifth day of the menstrual cycle. Ovulation stimulants included letrozole (Furui, China) and human menopausal gonadotropin (HMG) (Lizhu, China). hCG (Lizhu, China) was administered when the leading follicle reached 18 mm in diameter. The days of ovulation and FET were identical to those of women under NC FET protocol.

For women under the AC FET protocol, endometrial preparation was initiated with oral estradiol valerate (Progynova; Bayer Schering Pharma AG) at a daily dose of 4 mg from day 5 of menstrual cycle. The serum progesterone level was measured. A transvaginal ultrasound was performed 10–12 days later, provided that the endometrial thickness reached ≥7 mm, the serum progesterone level was <1.5 ng/mL, and vaginal progesterone soft capsules (0.2 g tid, Utrogestan, Besins, France) or vaginal progesterone gel (90 mg three times a day (qd), Crinone, Serono, Hertfordshire, UK) was commenced. FET was scheduled on days 4 and 6 of progesterone supplementation for cleavage stage embryos and blastocyst stage embryos, respectively.

All women received oral progesterone (10 mg tid; Dydrogesterone, Abbott Biologicals B.V., Amsterdam, Netherlands) and vaginal progesterone (Progesterone gel 90 mg qd, Crinone, Serono, Hertfordshire, UK; or progesterone soft capsules 0.2 g three times a day (tid), Utrogestan, Besins, France) simultaneously after FET. For women who underwent AC FET, exogenous estrogen was reduced after the confirmation of clinical pregnancy at 28 days after embryo transfer. The luteal support was maintained until week 10 of gestation.

### Definitions and outcomes

The primary outcome was the risk of GH, whereas the secondary outcomes were some other perinatal risks and neonatal risks. Other perinatal risks included stillbirth, preterm birth (<37 weeks of gestation), and gestational diabetes mellitus (GDM). GDM is defined as the onset or first recognition of carbohydrate intolerance during pregnancy (19, 20). To diagnose polycystic ovary syndrome (PCOS), the modified Rotterdam criteria were used, including menstrual abnormalities (irregular uterine bleeding, oligomenorrhea, or amenorrhea) combined with either hyperandrogenism or polycystic ovaries, as validated in our Chinese population. Stillbirth was defined as the absence of signs of life at or after 28 weeks of gestation. Neonatal risks included low birth weight (<2,500 g) and macrosomia (birth weight ≥4,000 g) (21).

### Confounding variables

Potential correlated factors of GH such as patient baseline demographical characteristics, ART treatment procedure, and pregnancy complications were also collected for all participants, including female age at OPU, female age at FET, male age, infertility duration, main etiology of infertility (tubal factor, ovarian factor, male factor, other reasons, or unknown reason), PCOS history (with or without), antral follicle count (AFC), parity (0 or ≥1), stimulation protocol (agonist protocol, antagonist protocol, or other protocols), insemination method, FET regimen (AC, NC, or OS FET), number of embryos transferred, number of good quality embryos transferred (0 or ≥1), type of embryo transferred (D3 cleavage-stage embryo, D5, or D6 blastocyst-stage embryo), and endometrial thickness.

### Statistical analysis

Continuous variables are presented as mean ± standard deviation. The Kolmogorov–Smirnov test was used to determine whether continuous variables were normally distributed. One-way analysis of variance or the Kruskal–Wallis test was used to compare the differences among the groups. Categorical variables were presented as count and proportion and were compared by Pearson’s chi-square test or Fisher’s exact test.

Generalized linear model was used to evaluate the associations between pre-gravid BMI, reproductive, and perinatal outcomes. Furthermore, we explored the relationship between pre-gravid BMI and GH with a generalized additive model. If non-linear correlation was observed, then a two-piecewise linear regression model was used to calculate the threshold effect of the pre-gravid BMI on HP in terms of the smoothing plot. For multivariate analyses, logistic regression was utilized.

All the analyses were performed with the statistical software packages R (http://www.R-project.org, The R Foundation) and EmpowerStats (http://www.empowerstats.com, X&Y Solutions, Inc., Boston, MA). The level of significance was set at *P* < 0.05.

## Results

### Composition of participants and baseline characteristics

A total of 18,624 patients underwent FET between January 2018 and December 2022 at our center. The data of total population are shown in **Supplementary Table 1**. Among them, 7,502 participants met the inclusion criteria (**Supplementary Figure 1**).

As shown in **Table 1**, of the 7,502 participants, 5,403 (72.02%) participants were categorized into the normal BMI group, 1,622 (21.62%) participants were categorized into the overweight group, and 477 (6.36%) participants were categorized into the obese group according to the BMI definitions of the WGOC. There were significant differences among the three groups in all the baseline characteristics including female age at OPU, female age at FET, male age, infertility duration, cause of infertility, proportion of patients with PCOS, and AFC (all *P* < 0.05).

**Table 1:** Characteristics of the patients at baseline; BMI groups were based on WGOC.

	Normal (n = 5,403)	Overweight (n = 1,622)	Obese (n = 477)	*P*-value
Female age at OPU (years)	30.15 ± 3.79	30.67 ± 4.18	29.82 ± 4.07	<0.001^a,b^
Female age at FET (years)	30.85 ± 3.77	31.31 ± 4.21	30.42 ± 4.11	<0.001^a,b,c^
Male age (years)	31.75 ± 4.53	32.28 ± 4.83	31.77 ± 4.43	<0.001^a,b^
Infertility duration (years)	3.25 ± 2.31	3.62 ± 2.56	3.97 ± 2.53	<0.001^a,b,c^
Cause of infertility				<0.001^b,c^
Tubal factor, n (%)	2512 (46.49%)	704 (43.40%)	179 (37.53%)	
Ovulation factor, n (%)	358 (6.63%)	210 (12.95%)	65 (13.63%)	
Male factor, n (%)	1,065 (19.71%)	245 (15.10%)	69 (14.47%)	
Unknown reason, n (%)	253 (4.68%)	79 (4.87%)	23 (4.82%)	
Other factors, n (%)	1,215 (22.49%)	384 (23.67%)	141 (29.56%)	
With PCOS	500 (9.25%)	311 (19.17%)	137 (28.72%)	<0.001^a,b,c^
Antral follicle count (n)	12.87 ± 6.24	14.21 ± 7.00	15.84 ± 7.51	<0.001^a,b,c^
Parity				<0.001^a,b^
None, n (%)	4,692 (87.26%)	1,337 (82.79%)	421 (88.82%)	
≥1, n (%)	685 (12.74%)	278 (17.21%)	53 (11.18%)	

Data are presented as mean ± SD or n (%); statistical significance is defined as P < 0.05; ^a,^ there is a significant difference between normal group and overweight group; ^b,^ there is a significant difference between overweight group and obese group; ^c,^ there is a significant difference between normal group and obese group; BMI, body mass index; WGOC, Working Group on Obesity in China; OPU, oocyte pickup; FET, frozen-thawed embryo transfer; PCOS, polycystic ovarian syndrome.

On the basis of the WHO classification, the comparisons of baseline characteristics are shown in **Supplementary Table 2**.

### Pre-gravid BMI and GH

As shown in **Table 2**, the incidence of GH in the study population was 6.15% (461/7,502). GH risk in the obese group was 15.55%, which was significantly higher than that of the normal BMI group (4.68%, *P* < 0.001) and the overweight group (8.26%, *P* < 0.001). Logistic regression models were generated to determine the effects of pre-gravid overweight and obesity on GH. After adjusting for infertility duration, cause of infertility, female age at OPU, and FET, pre-gravid overweight and obesity were significantly associated with higher GH risk (OR, 1.77; 95% CI, 1.41–2.20; *P* < 0.001; OR, 3.69; 95% CI, 2.77–4.91; *P* < 0.001) (**Table 3**).

**Table 2:** IVF/ICSI cycle features and outcomes.

	Normal (n = 5,403)	Overweight (n = 1,622)	Obese (n = 477)	*P*-value
Stimulation protocol				0.755
Agonist protocol, n (%)	3,528 (65.30%)	1,075 (66.28%)	320 (67.09%)	
Antagonist protocol, n (%)	1,494 (27.65%)	435 (26.82%)	130 (27.25%)	
Other, n (%)	381 (7.05%)	112 (6.91%)	27 (5.66%)	
Insemination method				0.514
IVF, n (%)	3,971 (73.50%)	1,215 (74.91%)	350 (73.38%)	
ICSI, n (%)	1,432 (26.50%)	407 (25.09%)	127 (26.62%)	
PGT, n (%)	237 (4.39%)	60 (3.70%)	25 (5.24%)	0.279
FET regimen				<0.001^a,c^
Artificial cycle, n (%)	4,350 (80.51%)	1,373 (84.65%)	419 (87.84%)	
Natural cycle, n (%)	1,019 (18.86%)	240 (14.80%)	58 (12.16%)	
Ovarian stimulation cycle, n (%)	34 (0.63%)	9 (0.55%)	0 (0.00%)	
No. of embryos transferred (n)				0.213
1	3,512 (65.00%)	1,030 (63.50%)	329 (68.97%)	
2	1,879 (34.78%)	589 (36.31%)	148 (31.03%)	
3	12 (0.22%)	3 (0.18%)	0 (0.00%)	
No. of good quality embryo transfer				0.408
None, n (%)	1,284 (23.76%)	411 (25.34%)	112 (23.48%)	
≥1 high quality embryo, n (%)	4,119 (76.24%)	1,211 (74.66%)	365 (76.52%)	
Type of embryo transferred				0.501
D3 cleavage-stage embryo, n (%)	1,207 (22.34%)	373 (23.00%)	107 (22.43%)	
D5 blastocyst-stage embryo, n (%)	3,843 (71.13%)	1,163 (71.70%)	341 (71.49%)	
D6 blastocyst-stage embryo, n (%)	353 (6.53%)	86 (5.30%)	29 (6.08%)	
Endometrial thickness (mm)	10.50 ± 1.74	10.67 ± 1.76	10.67 ± 1.84	<0.001^a^
Still birth rate, n (%)	4 (0.07%)	5 (0.31%)	0 (0.00%)	0.043^a^
Preterm birth rate, n (%)	416 (7.73%)	188 (11.62%)	77 (16.18%)	<0.001^a,b,c^
Live birth rate, n (%)	5380 (99.57%)	1,613 (99.45%)	476 (99.79%)	0.579
Gestational hypertension, n (%)	253 (4.68%)	134 (8.26%)	74 (15.55%)	<0.001 ^b,c^
Gestational diabetes mellitus, n (%)	306 (5.67%)	151 (9.31%)	68 (14.29%)	<0.001
Mode of delivery				<0.001^a,b,c^
Vaginal delivery, n (%)	1,353 (25.13%)	328 (20.27%)	65 (13.66%)	
Cesarean section, n (%)	4,031 (74.87%)	1290 (79.73%)	411 (86.34%)	
Birth weight (Kg)	3.34 ± 0.50	3.38 ± 0.57	3.34 ± 0.62	0.022^a^
Low birth weight, n (%)	236 (4.38%)	93 (5.77%)	36 (7.56%)	0.002^a,c^
Macrosomia, n (%)	444 (8.25%)	202 (12.52%)	59 (12.39%)	<0.001^a,c^
Birth height (cm)	50.03 ± 1.61	50.03 ± 1.97	49.80 ± 2.60	0.031^b,c^

Data are presented as mean ± SD or n (%); statistical significance is defined as P < 0.05; IVF, in vitro fertilization; ICSI, intracytoplasmic sperm injection; PGT, preimplantation genetic testing; FET, frozen-thawed embryo transfer; ^a,^ there is a significant difference between normal group and overweight group; ^b,^ there is a significant difference between overweight group and obese group; ^c,^ there is a significant difference between normal group and obese group.

**Table 3:** Effects of pre-gravid BMI on GH.

	Adjusted model I			Adjusted model II		
	OR	95% CI	*P*-value	OR	95% CI	*P*-value
Normal	Ref			Ref		
Overweight	1.77	1.41, 2.20	<0.001	1.82	1.458, 2.26	<0.001
Obese	3.69	2.77, 4.91	<0.001	3.46	2.59, 4.61	<0.001

Infertility duration, cause of infertility, female age at OPU, and FET were adjusted in model I; FET regimen was adjusted in model II.

**Figure 1:**
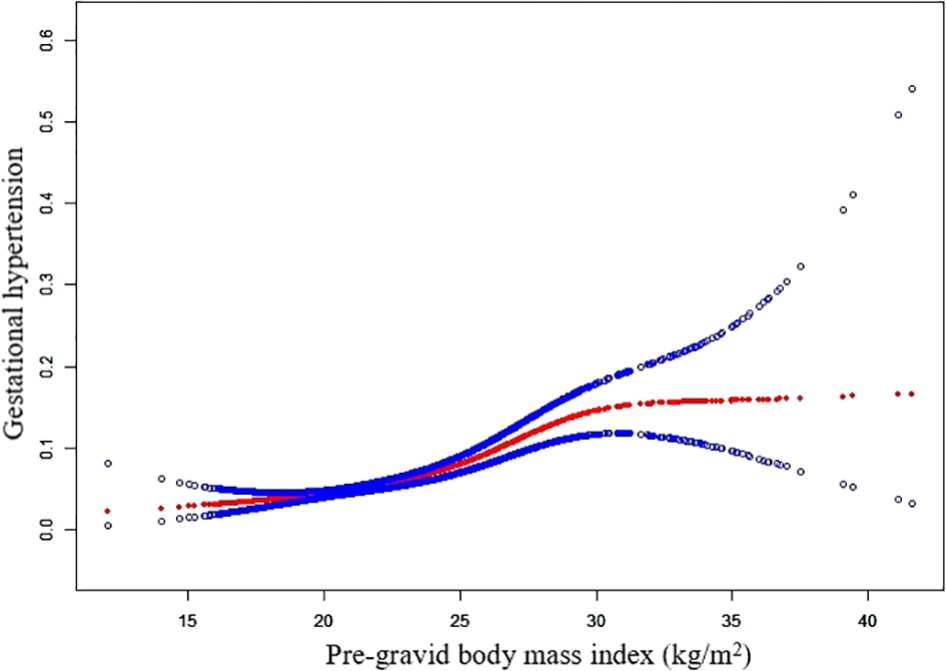
Non-linear relationship between pre-gravid BMI and GH.

Because pre-gravid BMI is a continuous variable, the analyses of non-linear relationships are necessary. **Figure 1** shows that the relationship between pre-gravid BMI and GH was non-linear (after adjusting baseline characteristics and FET regimen). By two-piecewise linear regression model, the pre-gravid BMI inflection point was calculated to be 28.6 kg/m^2^. The risk of GH decreased with pre-gravid BMI level up to the turning point of BMI = 28.6 kg/m^2^ (OR, 1.164; 95% CI, 1.124–1.205; *P* < 0.001). When pre-gravid BMI was ≥28.6 kg/m^2^, it was not associated with an increasing risk of HDP (OR, 0.965; 95% CI, 0.858–1.085; *P* = 0.553) (**Supplementary Table 3**).

### Pre-gravid BMI and other perinatal outcomes

As shown in **Table 2**, the stillbirth rate of the overweight group was 0.31%, which was significantly higher than that of the normal BMI group (0.07%, *P* < 0.05). Preterm birth rates (7.73%, 11.62%, and 16.18%) and cesarean section rates (74.87%, 79.73%, and 86.34%) increased with increase of BMI, and there were significant differences in the three groups (*P* < 0.05). The incidence of GDM in the normal BMI group was 5.67%, which was significantly lower than that of the overweight group (9.31%) and the obese group (14.29%) (*P* < 0.001). There was no significant difference among the three groups in live birth rates.

### Pre-gravid BMI and neonatal outcomes

Birth weight of neonates in the overweight group (3.38 ± 0.57) was significantly higher than that of the normal BMI group (3.34 ± 0.50, *P* < 0.05). Low birth weight rate of neonates in the normal BMI group (4.38%) was significantly lower than that of the overweight group (5.77%) and the obese group (7.56%) (*P* < 0.05). Moreover, macrosomia rate of neonates in the normal BMI group (8.25%) was significantly lower than that of the overweight group (12.52%) and the obese group (12.39%) (*P* < 0.05). Birth height of the obese group (49.80 ± 2.60) was significantly shorter than that of the normal BMI group (50.03 ± 1.61) and the overweight group (50.03 ± 1.97) (*P* < 0.05).

## Discussion

In this large cohort study among infertile women who achieved singleton pregnancy following FET, we found that pre-gravid overweight and obesity may be indications of GH. When pre-gravid BMI was <28.6 kg/m^2^, GH risk may be reduced with the decrease of BMI.

ART is a known risk factor of GH (10). GH rates were around 4.2% in non-ART and could reach 5.6% in ART singleton pregnancy (22). In our study, total GH rate reached 6.15%, which was slightly higher than that in previous reports. The reason is that our study cohort included patients who underwent FET, which was associated with higher GH risk as compared with fresh embryo transfer (9, 10). However, the mechanism for this phenomenon is unclear. GH rates in the obese group were 15.55% in this study. The combination of high BMI and FET was particularly detrimental.

Asian populations have different associations between BMI, body fat percentage, and health risks than European populations. In addition, our study population was Chinese pregnant women (23). Thus, we applied both WHO and WGOC definitions of overweight and obesity in this study.

Zhang et al. identified the effect of BMI on pregnancy-related complications (including pregnancy-induced hypertension and PE) in FET cycles in 2019 (24). However, they did not analyze the incidence of pregnancy-induced hypertension and PE in overweight and obese FET pregnancies, respectively. In 2013, their team reported the impact of maternal BMI on singleton pregnancies after FET. Concerning obesity being a “higher risk” compared with overweight, their study showed an association between obesity, PE, and eclampsia in FET pregnancies (11). However, overweight was more prevalent and should not be ignored. According to the latest national prevalence estimates for 2015–2019, based on Chinese criteria, overweight was 34.3% and obesity was 16.4% in adults (25). Moreover, their study analyzed only PE and eclampsia. GH is an important subtype of HDP. Here, we analyzed the impact of pre-gravid BMI on GH in singleton pregnancy following FET, which makes our study different from the previous studies.

Consistent with previous findings, the proportion of patients diagnosed with PCOS increased successively in the normal BMI group, the overweight group, and the obese group, with a significant difference between the three groups (11). PCOS is an endocrine and metabolic disorder, which is an independent risk factor for HDP (OR, 1.48; 95% CI, 1.48–1.60) (26). Metabolic characteristics of PCOS include obesity, insulin resistance, and type 2 diabetes mellitus. These factors may extend into pregnancy and lead to increased risk of pregnancy complications such as GH and GDM and adversely affect neonatal outcomes (27). Therefore, in the present study, we observed significantly higher incidences of GDM, heavier birth weights, and taller birth heights in the overweight group and the obese group as compared with that in the normal BMI group.

Our results validate the association between pre-gravid obesity and neonatal outcomes. Pre-gravid obesity before FET was associated with higher risks of low birth weight and macrosomia. Women preparing for FET should maintain a normal BMI to lower the chances of neonates with low birth weight and macrosomia.

Most studies have focused on the effects of pre-gravid BMI on GH and strongly recommended that women with obesity should make efforts to lose weight before IVF or FET. However, the linear relationship between pre-gravid BMI and the risk of GH was unknown. In our present study, we established a linear model to evaluate the effects of pre-gravid BMI on the risk of GH based on our FET cycle data. We found that, before the inflection point of BMI = 26.8 kg/m^2^, the risk of GH may increase by 16.4% with each unit increase in maternal BMI. In women with BMI>26.8 kg/m^2^, the risk of GH did not increase significantly with increasing BMI. However, the number of pregnancies with BMI >26.8 kg/m^2^ in our present study was insufficient. On the basis of these conclusions, clinicians and patients in China could better predict the risk of GH after FET and set personal weight loss goals before FET.

First, the major strength of this study is the large cohort size from a single-center, in which practice consistency can be assured. Controlled OS, IVF protocols, and laboratory conditions remained homogeneous. Second, patients were categorized according to the WGOC and the WHO classification, respectively, which makes the results more referable. Third, rigid inclusion criteria were set: Older women (>40 years) and multiple pregnancies were excluded to better demonstrate the adverse effects of pre-gravid overweight and obesity on GH.

This study had several limitations. First, this a single-center retrospective cohort study, and studies often cannot adequately control for all confounders. Second, we failed in exact classification of patients into HDP categories after telephone follow-up. We followed up patients by telephone at 1 month after their expected date of delivery, which may have resulted in recall bias.

## Conclusions

Pre-gravid overweight and obesity are associated with higher GH risk among singleton pregnancy following FET. Before the turning point of BMI = 26.8 kg/m^2^, the risk of GH may increase by 16.4% with each one-unit increment of maternal BMI. However, further large-scale, prospective, randomized controlled trials with a longer follow-up are required to verify the effect of pre-gravid BMI in women who achieved singleton pregnancy after FET.

## Data availability statement

The original contributions presented in the study are included in the article/**Supplementary Material**. Further inquiries can be directed to the corresponding author.

## Ethics statement

The studies involving humans were approved by Northwest Women’s and Children’s Hospital. The studies were conducted in accordance with the local legislation and institutional requirements. The participants provided their written informed consent to participate in this study.

## Author contributions

LF: Writing – original draft. NL: Investigation, Resources, Supervision, Validation, Visualization, Writing – review & editing. XM: Data curation, Funding acquisition, Writing – original draft. PQ: Data curation, Formal Analysis, Writing – original draft. JS: Project administration, Resources, Supervision, Writing – review & editing.
