# Supplementary material for: Pre-gravid body mass index is associated with a higher risk of gestational hypertension in singleton pregnancy following frozen-thawed embryo transfer
Source: Front Endocrinol (Lausanne). 2023 Oct 16;14:1258530. doi: 10.3389/fendo.2023.1258530 (PMC10614010; doi:10.3389/fendo.2023.1258530)
Supplement: Supplementary file 1 [file Table_1.docx]

Supplementary Table 1. Baseline characteristics, IVF/ICSI cycle features and reproductive outcomes of total cohort.

|  | Normal (n=13161) | Overweight (n=4136) | Obese (n=1207) | Missing BMI (n=120) | *P* Value |
| --- | --- | --- | --- | --- | --- |
| Female age at OPU (y) | 31.13 ± 4.63 | 31.70 ± 4.91 | 30.64 ± 4.66 | 30.28 ± 5.37 | <0.001 |
| Female age at FET (y) | 31.82 ± 4.57 | 32.35 ± 4.89 | 31.22 ± 4.66 | 34.04 ± 4.39 | <0.001 |
| Male age (y) | 32.67 ± 5.27 | 33.25 ± 5.62 | 32.39 ± 4.96 | 32.67 ± 6.47 | <0.001 |
| Infertility duration (y) | 3.39 ± 2.62 | 3.74 ± 2.82 | 4.03 ± 2.61 | 3.39 ± 2.64 | <0.001 |
| Cause of infertility |  |  |  |  | <0.001 |
| Tubal factor, n (%) | 5998 (45.57%) | 1756 (42.46%) | 452 (37.45%) | 14 (11.67%) |  |
| Ovulation factor, n (%) | 940 (7.14%) | 479 (11.58%) | 199 (16.49%) | 1 (0.83%) |  |
| Male factor, n (%) | 2370 (18.01%) | 607 (14.68%) | 169 (14.00%) | 3 (2.50%) |  |
| Unknown reason, n (%) | 685 (5.20%) | 212 (5.13%) | 45 (3.73%) | 1 (0.83%) |  |
| Other factors, n (%) | 3168 (24.07%) | 1082 (26.16%) | 342 (28.33%) | 101 (84.17%) |  |
| With PCOS | 1014 (7.70%) | 658 (15.91%) | 333 (27.59%) | 4 (3.33%) | <0.001 |
| Antral follicle count (n) | 11.85 ± 6.31 | 12.98 ± 7.07 | 14.83 ± 7.71 | 8.33 ± 4.50 | <0.001 |
| Parity |  |  |  |  | <0.001 |
| None, n (%) | 10821 (82.61%) | 3269 (79.38%) | 1006 (84.04%) | 20 (76.92%) |  |
| ≥1, n (%) | 2095 (15.99%) | 746 (18.12%) | 173 (14.45%) | 5 (19.23%) |  |
|  | 172 (1.31%) | 96 (2.33%) | 17 (1.42%) | 1 (3.85%) |  |
|  | 11 (0.08%) | 7 (0.17%) | 1 (0.08%) | 0 (0.00%) |  |
| Insemination method |  |  |  |  | 0.330 |
| IVF, n (%) | 9700 (73.70%) | 3082 (74.52%) | 889 (73.65%) | 96 (80.00%) |  |
| ICSI, n (%) | 3461 (26.30%) | 1054 (25.48%) | 318 (26.35%) | 24 (20.00%) |  |
| PGT | 408 (3.10%) | 103 (2.49%) | 35 (2.90%) | 7 (5.83%) | 0.057 |
| FET regimen |  |  |  |  | <0.001 |
| Artificial cycle | 10760 (81.76%) | 3568 (86.27%) | 1071 (88.73%) | 95 (79.17%) |  |
| Natural cycle | 2343 (17.80%) | 552 (13.35%) | 135 (11.18%) | 25(20.83%) |  |
| Ovarian stimulation cycle | 58 (0.44%) | 16 (0.39%) | 1 (0.08%) | 1 (0.83%) |  |
| No. of embryos transferred (n) |  |  |  |  | 0.007 |
| 1 | 7685 (58.39%) | 2327 (56.26%) | 747 (61.89%) | 76 (63.33%) |  |
| 2 | 5428 (41.24%) | 1798 (43.47%) | 458 (37.95%) | 43 (35.83%) |  |
| 3 | 48 (0.36%) | 11 (0.27%) | 2 (0.17%) | 1 (0.83%) |  |
| Good quality embryo transfer |  |  |  |  | <0.001 |
| None, n (%) | 4284 (32.55%) | 1457 (35.23%) | 400 (33.14%) | 54 (45.00%) |  |
| ≥1 high quality embryo, n (%) | 8877 (67.45%) | 2679 (64.77%) | 807 (66.86%) | 66 (55.00%) |  |
| Type of embryo transferred |  |  |  |  | 0.632 |
| D3 cleavage-stage embryo, n (%) | 4104 (31.18%) | 1335 (32.28%) | 375 (31.07%) | 40 (33.33%) |  |
| D5 blastocyst-stage embryo, n (%) | 8095 (61.51%) | 2521 (60.95%) | 735 (60.89%) | 73 (60.83%) |  |
| D6 blastocyst-stage embryo, n (%) | 962 (7.31%) | 280 (6.77%) | 97 (8.04%) | 7 (5.83%) |  |
| Endometrial thickness (mm) | 10.37 ± 1.81 | 10.58 ± 1.84 | 10.62 ± 1.89 | 10.29 ± 1.82 | <0.001 |
| Clinical pregnancy rate, n (%) | 7755 (58.92%) | 2450 (59.24%) | 731 (60.56%) | 71 (59.17%) | 0.530 |
| Miscarriage before 20 weeks of pregnancy, n (%) | 1278 (16.48%) | 532 (21.71%) | 172 (23.53%) | 11 (15.49%) | <0.001 |
| Ongoing pregnancy rate | 6406 (48.67%) | 1904 (46.03%) | 554 (45.90%) | 59 (49.17%) | 0.004 |
| Stillbirth rate, n (%) | 4 (0.06%) | 5 (0.26%) | 0 (0.00%) | 0 (0.00%) | 0.074 |
| Preterm birth rate, n (%) | 1057 (16.55%) | 371 (19.53%) | 130 (23.51%) | 12 (20.34%) | <0.001 |
| Live birth rate, n (%) | 6383 (48.50%) | 1895 (45.82%) | 553 (45.82%) | 59 (49.17%) | 0.004 |
| Gestational hypertension, n (%) | 344 (5.37%) | 181 (9.50%) | 85 (15.37%) | 0 (0.00%) | <0.001 |
| Gestational diabetes mellitus, n (%) | 355 (5.54%) | 167 (8.77%) | 74 (13.38%) | 1 (1.69%) | <0.001 |

Note: Data are presented as mean ± SD or n (%); Statistical significance is defined as *P*<0.05; OPU=Oocyte pick up; FET=Frozen thawed embryo transfer; PCOS= Polycystic ovarian syndrome; IVF=*in vitro* fertilization; ICSI=intracytoplasmic sperm injection; PGT=Pre-implantation genetic testing.

Supplementary Table 2. Characteristics of the patients at baseline, BMI groups were based on definitions of WHO.

|  | Normal (n=6029) | Overweight (n=1261) | Obese (n=212) | *P* value |
| --- | --- | --- | --- | --- |
| Female age at OPU (y) | 30.23 ± 3.84 | 30.46 ± 4.18 | 29.30 ± 4.02 | <0.001 |
| Female age at FET (y) | 30.92 ± 3.81 | 31.09 ± 4.23 | 29.93 ± 4.03 | <0.001 |
| Male age (y) | 31.25 ± 4.43 | 31.25 ± 4.43 | 31.25 ± 4.43 | 0.009 |
| Infertility duration (y) | 3.28 ± 2.35 | 3.71 ± 2.50 | 3.97 ± 2.54 | <0.001 |
| Cause of infertility |  |  |  | <0.001 |
| Tubal factor, n (%) | 2796 (46.38%) | 527 (41.79%) | 72 (33.96%) |  |
| Ovulation factor, n (%) | 427 (7.08%) | 176 (13.96%) | 30 (14.15%) |  |
| Male factor, n (%) | 1163 (19.29%) | 186 (14.75%) | 30 (14.15%) |  |
| Unknown reason, n (%) | 282 (4.68%) | 65 (5.15%) | 8 (3.77%) |  |
| Other factors, n (%) | 1361 (22.57%) | 307 (24.35%) | 72 (33.96%) |  |
| With PCOS | 596 (9.89%) | 290 (23.00%) | 62 (29.25%) | <0.001 |
| Antral follicle count (n) | 12.95 ± 6.29 | 14.79 ± 7.23 | 16.23 ± 7.61 | <0.001 |
| AMH (ng/mL) | 3.43 ± 3.30 | 3.41 ± 3.47 | 3.11 ± 2.71 | 0.925 |
| Parity |  |  |  | 0.214 |
| None, n (%) | 5195 (86.55%) | 1067 (85.16%) | 188 (89.10%) |  |
| ≥1, n (%) | 807 (13.45%) | 186 (14.84%) | 23 (10.90%) |  |

Note: Data are presented as mean ± SD or n (%); Statistical significance is defined as *P*<0.05; a. There is a significant difference between normal group and overweight group; b. There is a significant difference between overweight group and obese group; c. There is a significant difference between normal group and obese group; WHO= World health organization; OPU=Oocyte pick up; FET=Frozen thawed embryo transfer; PCOS= Polycystic ovarian syndrome; AMH= Anti-mullerian hormone.

Supplementary Table 3 Threshold effect of pre-gravid BMI on GH

|  | Crude RR (95% CI), *P* value | | | Adjusted RR^d^ (95% CI), *P* value | | |
| --- | --- | --- | --- | --- | --- | --- |
| BMI <28.6^e^ | 1.15 | 1.11,1.19 | <0.0001 | 1.16 | 1.12,1.21 | <0.001 |
| BMI ≥28.6^e^ | 0.88 | 0.76,1.02 | 0.088 | 0.97 | 0.86, 1.09 | 0.553 |

Note: ^d^Model adjusted female age at OPU, Female age at FET, proportion of patients with PCOS, FET regimen, insemination method, infertility duration and couple’s education degree; ^e^Assessment of RR and 95% CI for every one-unit increment of BMI. BMI=body mass index; GH=gestational hypertension; RR=relative risk; CI=confidence interval.

.
