# Supplementary figures and images for: Pre-gravid body mass index is associated with a higher risk of gestational hypertension in singleton pregnancy following frozen-thawed embryo transfer
Source: Front Endocrinol (Lausanne). 2023 Oct 16;14:1258530. doi: 10.3389/fendo.2023.1258530 (PMC10614010; doi:10.3389/fendo.2023.1258530)

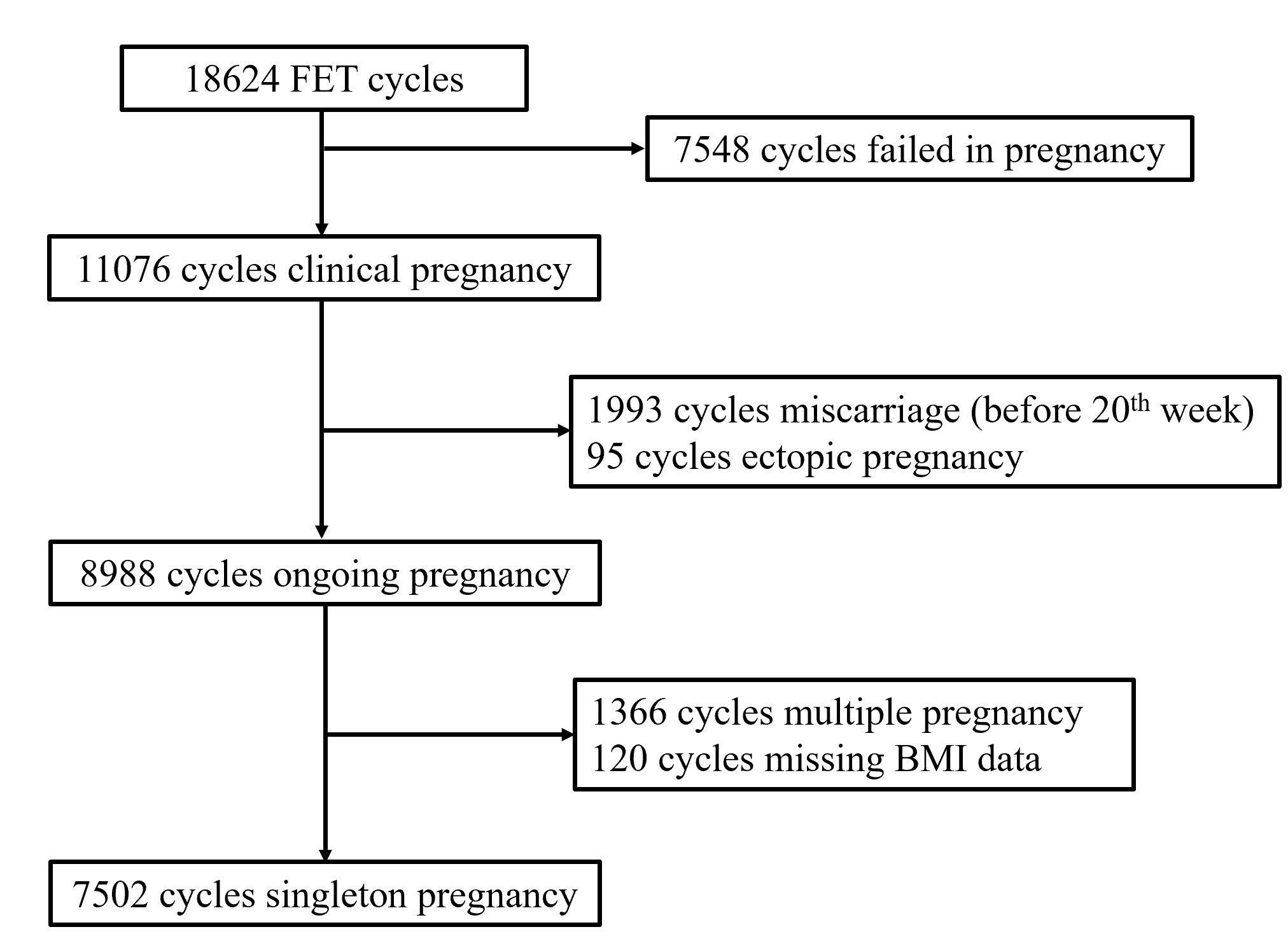

Supplement: Supplementary file 2 [file Image_1.jpeg]
